# Intraoperative ultrasound-guided compared to stereotactic navigated ventriculoperitoneal shunt placement: study protocol for a randomised controlled study

**DOI:** 10.1186/s13063-021-05306-5

**Published:** 2021-05-19

**Authors:** Severina Leu, Florian Halbeisen, Luigi Mariani, Jehuda Soleman

**Affiliations:** 1 Department of Neurosurgery, University Hospital of Basel, Spitalstrasse 21, 4031 Basel, Switzerland; 2 Basel Institute for Clinical Epidemiology & Biostatistics, University Hospital of Basel, Spitalstrasse 12, 4031 Basel, Switzerland; 3 Faculty of Medicine, University of Basel, Klingelbergstrasse 61, 4056 Basel, Switzerland

**Keywords:** Ventriculoperitoneal shunt, Ultrasound, Stereotaxic techniques, Hydrocephalus, Surgical technique, Randomised controlled trial

## Abstract

**Background:**

Ventriculoperitoneal shunt (VPS) placement is one of the most frequent neurosurgical procedures and the operation is performed in a highly standardised manner under maintenance of highest infection precautions. Short operation times are important since longer duration of surgery can increase the risk for VPS complications, especially infections. The position of the proximal ventricular catheter influences shunt functioning and survival. With freehand placement, rates of malpositioned VPS are still high. Several navigation techniques for improvement of shunt placement have been developed. Studies comparing these techniques are sparse. The aim of this study is to prospectively compare ultrasound (US) guided to stereotactic navigated shunt placement using optical tracking with the focus on operation time and efficiency.

**Methods:**

In this prospective randomised, single-centre, partially-blinded study, we will include all patients undergoing VPS placement in our clinic. The patients will be randomised into two groups, one group undergoing US-guided (US-G) and the other group stereotactic navigated VPS placement using optical tracking. The primary outcome will be the surgical intervention time. This time span consists of the surgical preparation time together with the operation time and is given in minutes. Secondary outcomes will be accuracy of catheter positioning, VPS dysfunction and need for revision surgery, total operation and anaesthesia times, and amount of intraoperative ventricular puncture attempts as well as complications, any morbidity and mortality.

**Discussion:**

To date, there is no prospective data available comparing these two navigation techniques. A randomised controlled study is urgently needed in order to provide class I evidence for the best possible surgical technique of this frequent surgery.

**Trial registration:**

Business Administration System for Ethical Committees (BASEC) 2019-02157, registered on 21 November 2019, https://www.kofam.ch/de/studienportal/suche/88135/studie/49552; clinicalTrials.gov: NCT04450797, registered on 30 June 2020.

## Administrative information

The order of the items has been modified to group similar items (see http://www.equator-network.org/reporting-guidelines/spirit-2013-statement-defining-standard-protocol-items-for-clinical-trials/).

**Table:** 

Title {1}	Intraoperative Ultrasound Guided compared to Stereotactic Navigated Ventriculoperitoneal Shunt Placement (NaVPS-Trial): Study Protocol for a Randomized Controlled Trial
Trial registration {2a and 2b}.	www.kofam.ch: BASEC 2019-02157, registration 11/21/2019 clinicalTrials.gov: NCT04450797, registration 06/30/2020
Protocol version {3}	Study Protocol Version 2, 01/11/2020.
Funding {4}	Gottfried and Julia Bangerter-Rhyner Foundation Research Foundation of the University Hospital of Basel, Switzerland
Author details {5a}	Severina Leu^1^, Florian Halbeisen^2^, Luigi Mariani^1,3^, Jehuda Soleman^1,3^ ^1^ Department of Neurosurgery, University Hospital of Basel, Spitalstrasse 21, 4031 Basel, Switzerland, severina.leu@unibas.ch ^2^ Basel Institute for Clinical Epidemiology & Biostatistics, University Hospital of Basel, Spitalstrasse 12, 4031 Basel, Switzerland ^3^ Faculty of Medicine, University of Basel, Klingelbergstrasse 61, 4056 Basel, Switzerland
Name and contact information for the trial sponsor {5b}	Prof. Dr. med. Luigi Mariani, Chair Department of Neurosurgery University Hospital of Basel, Spitalstrasse 21, 4031 Basel, Switzerland
Role of sponsor {5c}	The sponsor contributed to the conception and design of the study and he will be involved in the decision to submit the report for publication. The funding bodies have no role in the design of the study, in collection, analysis, and interpretation of data, or in writing or publishing the results.

## Introduction

### Background and rationale {6a}

Ventriculoperitoneal shunt (VPS) placement is one of the most frequent procedures in neurosurgical practice. The indications vary from normal pressure hydrocephalus in elderly patients to VPS dependency after subarachnoid haemorrhage, infection or trauma, mostly in younger patients [1–3].

This operation is done in a highly standardised manner with many steps taking place in an exactly defined order while maintaining highest infection precautions. Short operation and anaesthesia times are important since many patients requiring VPS placement are sick and prone for infections. There is data in the literature suggesting an association with longer duration of surgery and VPS complications, especially infections [4–6].

Insertion of VPS can be done using freehand technique or with the help of a navigation technique. VPS placement using optical stereotactic navigation has shown a high accuracy in catheter positioning, while the main limitations are that for referencing, the head of the patient needs to be fixed in a head holder and pins and the preoperative set-up can be time-consuming [7], leading to longer operation times. There are electromagnetic stereotactic navigation systems not requiring head fixation; however, the accuracy when used for neurosurgical procedures might be lower than for navigation systems where the head is fixed [8–12]. US-G VPS placement was described as a valid alternative to stereotactic navigation, while head fixation as well as preoperative planning and registration are not needed [13, 14].

The position of the proximal ventricular catheter is important since it influences possible malfunction of the VPS as well as shunt survival [15–18]. With freehand placement, rates of malpositioned VPS are still high (around 20%) [19]. It was previously shown that US-G proximal catheter placement is significantly more accurate than freehand placement and that the use of intraoperative guidance techniques reduces proximal shunt failure and lead to decrease in VPS revisions [20–22]. Stereotactically navigated VPS placement using optical tracking leads to high accuracy [23, 24]. A retrospective cohort study compared freehand, stereotactic navigated, and US-G VPS placement. There was no significant difference in accuracy of catheter placement between stereotactic guided and US-G placement, whereas both methods were superior to freehand placement [20].

Most of the clinical evidence regarding image-guided VPS placement derives from small retrospective cohort studies [14, 20–22]. To date, studies comparing US-G versus stereotactic navigation in a prospective randomised setting do not exist.

This study will prospectively compare US-G VPS placement to stereotactic navigation in a randomised controlled fashion with the surgical intervention time as the primary outcome.

Further, accuracy of catheter positioning, VPS dysfunction and need for revision surgery, total operation and anaesthesia times, and amount of intraoperative ventricular puncture attempts as well as complications, any morbidity and mortality will be compared as secondary outcomes between the two methods.

### Objectives {7}

With this study, we aim to prospectively evaluate the operating time and efficiency of US-G versus stereotactic navigated VPS placement. From the literature, we know that both methods have a similarly high accuracy in catheter positioning [20]. However, we think that time used for preparation and intraoperative set up is significantly different between the two methods and this might influence safety and morbidity of the procedure.

The main objective of this study is therefore to compare surgical intervention time between the groups

This time is composed of the preparation time of the surgery (patient positioning, installation and set-up of the navigation system) and the operation time and will be recorded for every operation in a standardised manner. We hypothesise that the operation time spent for US navigation will be shorter than the time spent for stereotactic navigation, affecting risk for VPS complications, cost efficiency, and anaesthesia time [4–6].

Secondary objectives of the study include:

- To compare operation time (time from start to end of neurosurgical part of the operation, “skin to skin”) and anaesthesia time.
- To compare catheter positioning based on postoperative imaging. Optimal catheter placement has been defined as free-floating within the CSF without touching the ventricle wall or septum pellucidum, and the tip of the catheter located at the foramen of Monro showing an optimal length of the catheter. Catheters that do not fulfil all criteria are defined as not optimally placed. Positioning of catheters will be further graded according to Yim et al. into grades I to IV (grade I, catheter terminates in the ipsilateral frontal horn; grade II, catheter terminates in contralateral frontal horn; grade III, catheter terminates in non-targeted CSF spaces; grade IV, catheter terminates in brain parenchyma) [17].
- To compare the rate of VPS dysfunction and need for revision surgery within 6 months of the study.
- To compare the amount of intraoperative puncture attempts of the ventricular system.
- To compare complication rates and mortality within 6 months of surgery.

### Trial design {8}

Prospective, randomised, controlled superiority trial. Randomisation is done into 2-arm parallel group with 1:1 allocation. This study is a phase III trial since both techniques are in use and licensed.

## Methods: participants, interventions, and outcomes

### Study setting {9}

Single-centre study, conducted at the Neurosurgical Department of the University Hospital of Basel, Switzerland.

### Eligibility criteria {10}

Inclusion criteria:

- Adult Patients undergoing VPS placement (≥18 years)
- Frontal or occipital shunt placement

Exclusion criteria:

- Emergency surgery if there is no time for installation of any navigation system
- Revision surgery using the same side and location
- Revision surgery addressing only some part of the shunt system (e.g*.* distal or proximal revision only)
- Ventriculoatrial and ventriculopleural shunt placement

### Who will take informed consent? {26a}

The investigators will explain to each participant the nature of the study, its purpose, the procedures involved, the expected duration, the potential risks and benefits, and any discomfort it may entail. All participants eligible for the study will be provided a participant information sheet and a consent form describing the study and providing sufficient information for them to make an informed decision about their participation in the study. Informed consent is obtained under usual circumstances one day preoperatively or directly before surgery in case of emergency surgery by the principal investigator (PI) or a designee that is adequately qualified to inform the participant about the study and to answer all the questions.

The study population includes vulnerable participants. If patients are not capable of judgement (e.g. dementia/neurologic state), an informed consent of the next of kin or an independent physician will be obtained. If, in case of emergency, the next of kin is not available, informed consent will be obtained from an independent physician on site. At a later timepoint, informed consent by the patient or his next of kin will be sought. If at any time the patient shows signs that he or she is not willing to participate in the study he/she will not be included in the study.

The patient information sheet and the consent form were submitted to the Competent Ethics Committee and were approved after reviewing. The participant will be given a copy of the signed document, while the original document will be retained as part of the study records.

### Additional consent provisions for collection and use of participant data and biological specimens {26b}

Separate consent is sought for possible subsequent use of collected data for future studies.

### Interventions

#### Explanation for the choice of comparators {6b}

The main aim of this study is to focus on the safety and feasibility of US-G VPS placement, with a special interest lying on the surgical intervention time (primary outcome). No preplanning, head fixation, or preoperative registration is needed. It is a real-time navigation method with direct visualisation of the correct catheter placement.

US itself is not known to have any harmful side effects and does not contain harmful radiation; therefore, it would be an optimal solution in the daily practice for routine use in all patients, especially in children and pregnant women.

Stereotactic navigated VPS placement using optical navigation is in use for many years. It has great accuracy even with slit ventricles [23, 24]. During navigation, pre-planned images can be seen without real-time correction, as opposed to US guidance. Preplanning and preoperative registration are mandatory for stereotactic navigation, and optical navigation requires also head fixation (as opposed to electromagnetic stereotactic navigation), possibly prolonging surgical intervention time and therefore leading to higher operating costs and longer duration of anaesthesia.

To date, no prospective data is available comparing US-G and stereotactic navigated VPS placement.

#### Intervention description {11a}

At our institution, we routinely insert a frontal VPS, while rarely an occipital VPS is placed. Since 2014, the abdominal shunt placement is performed by a visceral surgeon using the laparoscopic technique [25]. The “Kocher entry point” (measured at 11–12cm from Nasion and 3–3.5cm lateral to the midline) is used as the frontal entry point, the “Trigonum entry point” (measured at 4cm behind, and 5.5cm above the external auditory canal) is used as the occipital entry point. All catheters are connected to a burr-hole reservoir, a programmable valve (Codman Hakim® CERTAS^TM^ Plus, Integra LifeSciences, USA), and a distal peritoneal catheter.

##### US navigation group

There is no need for pre-planning, registration, or head fixation before the operation. After intubation, the head of the patient is placed on a horseshoe head holder slightly rotated to the contralateral side. Directly before the operation, the distal shunt parts (peritoneal catheter, valve, and burr hole reservoir) are connected to each other and fixed with sutures. After skin incision, a large burr hole is placed with a 14–11-mm high speed drill at 12cm from Nasion and 3cm lateral to the intended side of shunt placement. Lateral enlargement of the burr hole is done until passing of the catheter through the adapted guide-channel lateral to the US burr hole probe is possible. US-G (BK Medical 5000, bk medical Medizinische Systeme GmbH, Quickborn, Germany) VPS placement is done using a burr hole probe (type 9063 N11C5S, 11-5 MHz) onto which a sterile single-use guide channel, adaptable for different catheter diameters, is mounted.

Following the dural opening, real-time US navigation is done holding the US probe in the coronal plane, showing a cross-section through both frontal horns. Saline irrigation is applied to achieve better contrast. The foramen of Monro and choroid plexus are visualised, and the catheter is inserted along the inline trajectory aiming with the tip at the foramen of Monro. After positioning of the proximal catheter and functional control, the US probe is removed, and the catheter is shortened and connected to the distal shunt-parts. The peritoneal catheter is then implanted into the abdomen using the laparoscopic technique.

The physicians are already trained in the use of the US burr hole probe since it has been in use for many other operations, and VPS as well as external ventricular drain (EVD) placement before. The operation is performed by a trained neurosurgeon or by an advanced resident under supervision.

Patients in the US cohort need to have a computer tomography (CT) or magnetic resonance imaging not older than 90 days old (counted back from the operation day).

##### Stereotactic navigation group using optical navigation

Patient in the stereotactic group need to have a recent CT at admission. The exact location of the entry point, the trajectory, and the length of the catheter will be pre-planned on this CT scan at a Brainlab Workstation using cranial navigation software version 3.1. (Brainlab AG, Munich, Germany). After intubation, the head of the patient will be fixed in a Mayfield head clamp slightly rotated to the contralateral side of the intended VPS placement. Before starting the operation, the navigation will be registered and the incision will be marked at the pre-planned entry point. The distal shunt parts are also connected to each other directly before skin incision.

The ventricular catheter will be inserted with the navigated stylet (Brainlab AG, Munich, Germany) exactly along the pre-planned trajectory. After functional control, the catheter is cut in the precalculated length and connected to the distal shunt parts. The other steps of the operation are exactly the same as described above for US-G VPS placement.

The physicians are already trained in using the Brainlab navigation since it is in use for almost every craniotomy and for many other operations. The operation is performed by a trained neurosurgeon or by an advanced resident under supervision.

Patients in the stereotactic navigation cohort need to have a computer tomography (CT) or magnetic resonance imaging not older than 5 days old (counted back from the operation day).

#### Criteria for discontinuing or modifying allocated interventions {11b}

Patients will be withdrawn from the study in case of withdrawal of informed consent or non-compliance.

In patients with very small ventricles, the surgeon can decide to use stereotactic navigation instead of US navigation based on his individual judgement. Due to preoperative planning and necessity of a CT scan at admission, this crossover has to be done preoperatively.

Vice versa, there is the possibility to intraoperatively change from stereotactic navigation to US navigation in cases where a real-time navigation method is needed (e.g*.* in cases with distorted ventricular anatomy and unsuccessful placement using the stereotactic method).

#### Strategies to improve adherence to interventions {11c}

The PI or a designee is directly supervising the adherence to the randomised navigation technique.

#### Relevant concomitant care permitted or prohibited during the trial {11d}

There are no relevant concomitant care or interventions that are permitted or prohibited during the trial.

#### Provisions for post-trial care {30}

There are neither provisions foreseen for ancillary and post-trial care, nor compensation to those who suffer harm from trial participation.

### Outcomes {12}

#### Primary outcome: surgical intervention time

This time spans the time spent in the OR by the neurosurgeon, beginning with any intervention on the patient (e.g. positioning, head clamping) and ends when the neurosurgical part of the operation is finished. IT therefore consists of the preparation time and the operation time of the neurosurgical part of the operation. To minimise bias, the visceral surgery part of the operation (laparoscopically assisted intraperitoneal insertion of the distal catheter) will not be included within this time interval. These times are recorded for all operations in our department in a standardised manner by a person that is blinded for the study.

#### Secondary outcomes

*Operation time and anaesthesia time in minutes:* these times will be assessed on the operation day in a standardised manner by a blinded person

*Number of puncture attempts:* this will be assessed at the operation day by the operating surgeon.

*Catheter placement (optimal vs. not optimal, grades I to IV):* between the 2nd and 3rd postoperative day, a CT scan will be made and VPS positioning will be assessed. Optimal catheter placement is defined as free-floating within the cerebrospinal fluid (CSF) without touching the ventricle wall or septum pellucidum, and the tip of the catheter located at the foramen of Monro showing an optimal length of the catheter. Catheters that do not fulfil all criteria are defined as not optimally placed. Positioning of catheters will be further graded according to Yim et al. into grades I to IV (grade I, catheter terminates in the ipsilateral frontal horn; grade II, catheter terminates in contralateral frontal horn; grade III, catheter terminates in non-targeted CSF spaces; grade IV, catheter terminates intraparenchymally) [17]. Another CT scan is made in the second follow-up after 6 months. The assessment of the CT scans will be made by a blinded neuroradiologist.

*Volumetry of side ventricles pre- and postoperatively in cm*^*3*^ *(number and relative change):* this will be assessed on the pre- and postoperative CT scans by a blinded neuroradiologist.

*Evan’s Index (EI) pre- and postoperatively (number and relative change)* [26]*:* this will be assessed on the pre- and postoperative CT scans by a blinded neuroradiologist.

*Complications (infection, bleeding, complications associated with navigation method), mortality:* rate and detail of complications as well as mortality (including cause of mortality) will be assessed perioperatively, during the hospitalisation time and during the 1st and 2nd follow-up.

*VPS dysfunction (yes/no) and reason for dysfunction:* the rate of VPS dysfunction will be assessed perioperatively, during the hospitalisation time and during the 1st and 2nd follow-up.

*Revision surgery (yes/no) and reason for revision:* the rate of revision surgery as well as the indication for revision will be assessed directly postoperative, during the hospitalisation time and during the 1st and 2nd follow-up.

### Participant timeline {13}

Study flow chart/table of study procedures and assessments:

### Sample size {14}

- Total estimated patients, 130
- Estimated patients per group, 65

We used following formula to calculate the number of patients needed in each treatment arm [27]:

$$ \mathrm{n}=2{\upsigma}^2{\left\{\left[{Z}_{1-\alpha /2}+{Z}_{1-\upbeta}\right]/{\Delta }_A\right\}}^2 $$

σ^2^= sample variance; ${Z}_{1-\frac{\alpha }{2}}=$critical value from a standard normal distribution for *α*/2; *Z*_1 − *β*_=critical value from a standard normal distribution for *β*; ∆_*A*_=expected difference between treatment arms.

To correct for non-compliers, we then inflated the sample size using the drop-in and drop-out rates [27].

$$ {\mathrm{n}}_{\mathrm{c}\mathrm{orrected}}=\frac{\mathrm{n}}{{\left(1-{\mathrm{p}}_{\mathrm{c}}-{\mathrm{p}}_{\mathrm{t}}\right)}^2} $$

p_c_= drop-in rate; p_t_=drop-out rate

The sample size was estimated with the aim of showing a surgical intervention time reduction of 15 min. Based on data from a pilot study [14], we assumed a mean surgical intervention time of 63 min with a standard deviation (SD) of 28.65 min. The significance level was chosen to be 5%, while the power was chosen to be (1−*β*) = 80%. For the final estimation, we anticipating a drop-out rate of 1% and a surgeons’ non-adherence rate (change of treatment arm) of 5%. Sample size calculation was done using statistical software R [28].

### Recruitment {15}

Measurements are undertaken to screen all patients receiving a VPS operation due to several indications. The recruitment period lasts from the planning of the surgery until the surgery actually happens; this can be weeks for elective cases and hours for urgent cases. As soon as the operation is planned the patients are informed about the study and asked whether they would like to participate. Frequent training logs are undertaken to keep the team informed about the study and reminded for inclusion of eligible patients.

**Table:** 

Study periods	Screening admission	Treatment, intervention period			Follow-up	
Visit	1	2	3	4	5	6
Time (hour, day, week)	1 day preop.	Operation day	2–5 days post-op. (48–120h)	At discharge (approx. 7 days postop.)	6–8 weeks post-op.	6 months postop.
Patient information and informed consent	X					
Randomisation	X					
Demographics (age, sex)	X					
In-/exclusion criteria	X					
Neurologic examination	X		X	X	X	X
CT scan	(X stereotactic navigation group, 1 to 5 days preoperatively)		X (2nd to 5th day or earlier in case of neurologic symptoms)			X
Medical history	X					
Primary outcome (surgical intervention time)		X				
Secondary outcomes		X	X	X	X	X
VPS dysfunction		X	X	X	X	X
Operative revision and reasons		X	X	X	X	X
Operation and anaesthesia time		X				
Number of puncture attempts		X				
Complications		X	X	X	X	X
Hospitalisation time (days)				X		
Intensive care unit (ICU) time (days)				X		
Discharge destination				X		
Adverse events		X	X	X	X	X
Death		X	X	X	X	X

## Assignment of interventions: allocation

### Sequence generation {16a}

The allocation sequence is composed of computer-generated random numbers derived from the statistical programme R (R Core Team, Vienna, Austria) [29].

### Concealment mechanism {16b}

Randomisation will be performed using a stratified simple randomisation procedure as implemented in the electronic data capture (EDC) software REDCap (www.project-redcap.org, Vanderbilt University, Nashville, USA). An allocation ratio of 1:1 ensures a balance in sample size across both groups over time. The randomisation is stratified according to the age of the patients (under 40 years/over 40 years), thus leading to a balanced distribution of underlying causes of hydrocephalus between the groups.

Allocation concealment will be ensured, until the patient has been definitely recruited into the trial. The treating surgeon will then be informed about the assigned navigation method by the person performing the randomisation (PI or a designee). If the patient is randomised to the stereotactic navigated group, the patient will receive a new CT scan and the preoperative planning will be done on the Brainlab Work station usually 1 day preoperatively or in emergency operations directly before surgery.

### Implementation {16c}

The allocation sequence is composed of computer-generated random numbers derived from the statistical programme R [29]. Participant enrollment is done by the PI, the co-investigator, or the study coordinator using the electronic data capture software REDCap. Interventions are assigned in a randomised manner to the patients stratified according to age (under/over 40 years).

## Assignment of interventions: blinding

### Who will be blinded {17a}

Participants will be blinded for the navigation method they are randomised to. There is no possibility to blind the treating physician since he has to place the VPS with the selected navigation method and stereotactic navigation needs a pre-planning the day before the operation. The primary outcome (surgical intervention time) is recorded by a blinded person in a standardised manner for every operation. Some of the secondary outcomes on the pre- and postoperative CT scans are measured by a neuroradiologist that is blinded for the allocated navigation method.

### Procedure for unblinding if needed {17b}

Since the treating physician is not blinded for the allocated intervention, there is no need for specific unblinding procedures.

## Data collection and management

### Plans for assessment and collection of outcomes {18a}

#### Visit 1: Screening and admission

- Demographics (age, gender), height, weight, body mass index
- Neurologic examination (Glasgow Coma Scale (GCS), modified Rankin Scale (mRS), Glasgow Outcome Scale (GOS), neurological symptoms (headaches, vomitus, coma, gait disturbances, dementia, urinary incontinence, motor deficit, sensory deficit, aphasia, delir, others)
- Medical history (underlying condition causing hydrocephalus (Normal pressure hydrocephalus, subarachnoid haemorrhage, intraventricular haemorrhage, other type of bleeding, trauma, tumour, congenital, other), prior EVD, prior VPS, or prior head operations including details)
- CT scan (US group not older than 90 days, stereotactic navigation group not older than 5 days, measurements: EI, volumetry of side ventricles).

#### Visit 2: Operation day

- Primary outcome (surgical intervention time)
- Operation time, anaesthesia time
- Shunt side (right, left), shunt location (frontal, occipital), ventricular catheter length, number of surgeons, experience of main surgeon, shunt/valve manufacturer, type of valve (adjustable, non-adjustable), valve pressure, number of accessory incisions, number of puncture attempts
- Shunt dysfunction (yes/no)
- Revisions surgery (yes/no), indication for revision
- Complications including complication details
- Death including reason of death

#### Visit 3: 2nd to 5th postoperative day (48–120h postoperative)

- Neurologic examination (GCS, mRS, GOS, neurological symptoms (headaches, vomitus, coma, gait disturbances, dementia, urinary incontinence, motor deficit, sensory deficit, aphasia, delir), neurology better, headaches better, vomitus better, gait ataxia better, dementia better, urinary incontinence better)
- CT scan: catheter position (optimal vs. not optimal, grades I to IV), EI, volumetry of side ventricles, EI improvement, ventricle width reduction
- Shunt dysfunction including details (proximal/distal obstruction, proximal/distal dislocation, abdominal cause, dysfunction due to infection, disconnection, other)
- Revision surgery and indication for revision (bleeding, infection, obstruction, misplacement, disconnection, proximal/distal dislocation, other)
- Complications other than dysfunction (infection, bleeding, seizure, fracture, other)
- Death including reason of death

#### Visit 4: Discharge (approx. 7 days postoperatively)

- Neurologic examination (GCS, mRS, GOS, neurological symptoms (headaches, vomitus, coma, gait disturbances, dementia, urinary incontinence, motor deficit, sensory deficit, aphasia, delir), neurology better, headaches better, vomitus better, gait ataxia better, dementia better, urinary incontinence better)
- Shunt dysfunction including details (proximal/distal obstruction, proximal/distal dislocation, abdominal cause, dysfunction due to infection, disconnection, other)
- Revision surgery and indication for revision (bleeding, infection, obstruction, misplacement, disconnection, proximal/distal dislocation, other)
- Complications other than dysfunction (infection, bleeding, seizure, fracture, other)
- Duration of (postoperative) hospitalisation in days, duration of ICU stay in days
- Discharge destination (home, rehabilitation, nursing facility, other hospital, other)
- Death including reason of death

#### Visit 5: 1st follow-up (6–8 weeks postoperatively)

- Time of follow-up (date and number of days postoperative)
- Neurologic examination (GCS, mRS, GOS, neurological symptoms (headaches, vomitus, coma, gait disturbances, dementia, urinary incontinence, motor deficit, sensory deficit, aphasia, delir), neurology better, headaches better, vomitus better, gait ataxia better, dementia better, urinary incontinence better)
- Shunt dysfunction including details (proximal/distal obstruction, proximal/distal dislocation, abdominal cause, dysfunction due to infection, disconnection, other)
- Revision surgery and indication for revision (bleeding, infection, obstruction, misplacement, disconnection, proximal/distal dislocation, other)
- Complications other than dysfunction (infection, bleeding, seizure, fracture, other)
- Death including reason of death

#### Visit 6: 2nd follow-up (6 months postoperatively)

- Time of follow-up (date and number of days postoperative)
- Neurologic examination (GCS, mRS, GOS, neurological symptoms (headaches, vomitus, coma, gait disturbances, dementia, urinary incontinence, motor deficit, sensory deficit, aphasia, delir), neurology better, headaches better, vomitus better, gait ataxia better, dementia better, urinary incontinence better)
- CT scan: catheter position (optimal vs. not optimal, grades I to IV), EI, volumetry of side ventricles, EI improvement, ventricle width reduction
- Shunt dysfunction including details (proximal/distal obstruction, proximal/distal dislocation, abdominal cause, dysfunction due to infection, disconnection, other)
- Revision surgery and indication for revision (bleeding, infection, obstruction, misplacement, disconnection, proximal/distal dislocation, other)
- Complications other than dysfunction (infection, bleeding, seizure, fracture, other)
- Death including reason of death

### Plans to promote participant retention and complete follow-up {18b}

The two follow-ups in the study are foreseen after 6–8 weeks and after 6 months. These are the follow-ups all patients receive in our clinic after VPS implantation. Patients will be sent a letter with the exact time and place of the outpatient consultation several weeks beforehand.

Patient with deviation in intervention will be analysed in the intention-to-treat (ITT) analysis and the same outcome data will be collected as for the other patients.

### Data management {19}

The study data recorded in the case report form (CRF) will be transferred to a corresponding electronic CRF (e-CRF) by the study coordinator. The e-CRF will be implemented using the EDC software REDCap. The EDC software runs on a server maintained by the Information Technology Department at University Hospital Basel. REDCap data capture is accessible via a standard browser on a www-connected device. Password protection and user-right management ensures that only authorised persons can enter the EDC system to view, add, or modify data according to their permissions. An integrated audit trail system maintains a record of initial entries and changes (reason for change, date and time of change, and user identification). The database is backed up regularly according to the processes of the IT-department at University Hospital Basel.

The PI and co-investigator at the study site will be responsible for assuring that the data entered into the e-CRF is complete and accurate and that entry and updates are performed in a timely manner. All information recorded in the e-CRFs will be traceable to the source documents in the patient’s file and in the data source files.

All study data, including CRFs, Trial Master File, and informed consent forms will be archived for a minimum of 10 years after study termination or premature termination of the clinical trial. The study data will be archived in the research office of the Department of Neurosurgery University Hospital of Basel.

### Confidentiality {27}

Direct access to source documents will be permitted for purposes of monitoring. The participants name or other personal identifiable data are not recorded in the CRF as well as eCRF. Subjects’ confidentiality will be ensured by utilising unique identification numbers to correspond the data. Each participant will be coded by a number during the screening. After electronic enrolment to the study, the participant will be assigned with a unique personal study number (ID). All data collected for the study will be entered under the patient ID only.

The relation between the ID and participant’s name, address, date of birth, and screening number will be documented in a written register (“patient identification list”). The patient identification list will be stored in the research study office of the department of Neurosurgery, University Hospital of Basel.

The sponsor, PI, co-investigator, and study coordinator will have access to the protocol and dataset. Once the data of all subjects is transferred to the EDC system, the database will be locked and closed for further data entry. The complete study dataset is exported, encrypted, and transferred to the PI through a secured channel by the responsible Study Coordinator at University Hospital Basel.

### Plans for collection, laboratory evaluation, and storage of biological specimens for genetic or molecular analysis in this trial/future use {33}

There are no plans for collection, laboratory evaluation, and storage of biological specimens for genetic or molecular analysis in the current trial or for future use in ancillary studies.

## Statistical methods

### Statistical methods for primary and secondary outcomes {20a}

All analyses will be conducted using the statistical software package R [29]. Study findings will be reported according to the CONSORT guidelines [30]. Analysis will be conducted based on both the intention-to-treat and the per-protocol principles. A flowchart will describe the inclusion and follow-up of participants by study arm. Baseline characteristics will be described by study arm with summary statistics such as median and interquartile range or number and percentage; no formal testing between arms will be performed [31]. Outcomes will be described for each study arm using summary statistics.

The full analysis set will include all patients that were randomised. All statistical analyses will be performed on the full analysis set according to the ITT principle (i.e. all participants will be analysed on the basis of the intervention to which they were randomly allocated).

The per-protocol set will include all participants in the full analysis set who fulfilled the eligibility criteria, for whom the surgery was completed as planned in the study protocol, and for whom the measurement of the primary outcome is available.

#### Primary analysis

The primary outcome, surgical intervention time, will be assessed using a linear regression model, reporting adjusted mean differences between arms. The estimates will be reported with 95% confidence intervals (CI). The model will be adjusted for age, sex, body height, and BMI.

#### Secondary analyses

Operation time, anaesthesia time, volumetry of side ventricles, and EI will be analysed using linear regression models. Catheter placement, complications, mortality, and the need for revision surgery will be analysed using logistic regression models. Amount of ventricle puncture attempts will be analysed using Poisson regression model.

All estimates will be reported with 95% CI. All models will be adjusted for age, sex, body height, and BMI. We will compare each endpoint between the intervention and control arms.

### Interim analyses {21b}

There are no interim analyses planned for this study.

### Methods for additional analyses (e.g. subgroup analyses) {20b}

#### Safety analyses

Safety will be assessed via a rigorous and detailed examination of adverse events between the treatment group and the control group. Safety endpoints will be assessed using the full analysis set.

Safety endpoints are the following events: positioning of the catheter, number of ventricle puncture attempts, VPS dysfunction, revision rate, perioperative complications (bleeding, infection), complications due to head clamp, death, and coma.

Binary safety endpoints will be compared as number of events between the two groups (US and stereotactic navigation) using a logistic regression. To compare the number of ventricle puncture attempts per patient between the treatment and control arm, we will use a Poisson regression model.

### Methods in analysis to handle protocol non-adherence and any statistical methods to handle missing data {20c}

During the perioperative period, compliance to the randomised navigation method will be monitored by the PI. In cases of switched navigation method pre- or intraoperatively the reasons will be recorded. Finally, an ITT analysis will be performed.

Missing baseline and outcome data will be summarised by study arm. As outlined above, the primary analyses will be the ITT population. In the case of missing data or drop-outs, we may adjust for further baseline variables which are associated with missing outcome data [32] (which is analogous to performing multiple imputation in the case of a single endpoint). We may consider multiple imputation as sensitivity analyses if necessary.

### Plans to give access to the full protocol, participant-level data, and statistical code {31c}

We do not plan to give public access to the full protocol, participant-level dataset, or statistical code. The datasets analysed during the current study are available from the corresponding author on reasonable request.

## Oversight and monitoring

### Composition of the Coordinating Centre and Trial Steering Committee {5d}

The Study Coordinating Centre (Clinical Neurosurgical Research Centre, Basel) is part of the Trial Committee and has regular meetings (on a monthly basis) to discuss problems and/or difficulties within the study and their solutions as well as possible improvements. More urgent questions are discussed as needed, e.g. by mail contact or over the phone. For this trial, we did not set up a Trial Steering Committee since it is a single-centre study with a rather small cohort.

Of course, the trial is being monitored by an independent Monitor on a regular basis, which helps improve the study quality significantly, in addition to possible monitoring visits which do at time occur through the local Competent Ethics Committee (EKNZ, Basel).

### Composition of the data monitoring committee, its role, and reporting structure {21a}

The e-CRF and source data will be reviewed for completeness and accuracy through regular monitoring provided by the study site. The study staff will be available for the monitoring visits in order to give access to the study files and give any kind of support needed. The service will be provided by Heike Neddersen, clinical monitor of the Neurosurgery Clinic of the University Hospital of Basel.

### Adverse event reporting and harms {22}

Device deficiencies and all adverse events including all serious adverse events are collected, fully investigated, and documented in the source document and appropriate CRF during the entire study period, i.e. from patient’s informed consent until the last protocol-specific procedure, including a safety follow-up period. Documentation includes dates of event, treatment, resolution, assessment of seriousness and causal relationship to device and/or study procedure [International Organisation for Standardisation (ISO) 14155, 6.4.1 [33].]. All these hazards are reported to the Competent Ethics Committee by the PI within the prescribed time span.

### Frequency and plans for auditing trial conduct {23}

Inspections by regulatory authorities during the study or after the study is completed are performed to ensure proper study conduct and data handling procedures according to International Conference on Harmonisation-Good Clinical Practice (ICH-GCP) [34, 35] guidelines and regulatory requirements. Inspections may include verification of all source documents, e-CRF, site files, and a visual inspection of the study site. Direct access to all documents and sites involved in the study will be provided by the study staff members. In case of an announced inspection, immediate notification of the other party is necessary. All these processes will be done by persons that are independent from the PI or sponsor.

### Plans for communicating important protocol amendments to relevant parties (e.g. trial participants, ethical committees) {25}

Substantial amendments are only implemented after approval of the Competent Ethics Committee.

Under emergency circumstances, deviations from the protocol to protect the rights, safety, and well-being of human subjects may proceed without prior approval of the sponsor and the Competent Ethics Committee. Such deviations shall be documented and reported to the sponsor and the Competent Ethics Committee as soon as possible.

All non-substantial amendments are communicated to the Competent Ethics Committee within the Annual Safety Report.

## Dissemination plans {31a}

The trial is registered at clinicaltrials.gov (NCT04450797) and at www.kofam.ch. Publication of the final study results will be published in a top tier peer reviewed medical journal. There are no publication restrictions.

## Discussion

VPS placement is one of the most frequent neurosurgical procedures. The position of the proximal ventricular catheter is important for shunt function and survival. With freehand placement rates of malpositioned VPS are still high. Several navigation techniques for improvement of shunt placement have been developed. To date, there is no prospective data available for comparison of these navigation techniques. With this study, we focus on efficiency, feasibility and safety of the methods. This will hopefully improve our knowledge of the topic and refine the ideal therapeutic approach for these patients.

## Trial status

- Study Protocol Version 2, 01/11/2020
- Recruitment beginning: 02/2020
- Expected recruitment end: 10/2022

## Abbreviations

BASEC: Business Administration System for Ethical Committees
CT: Computer tomography
(e-)CRF: Electronic case report form
CSF: Cerebrospinal fluid
EDC: Electronic data capture
EI: Evans’ Index
EVD: External ventricular drain
GCS: Glasgow Coma Scale
GOS: Glasgow Outcome Scale
ICH-GCP: International Conference on Harmonisation-Good Clinical Practice
ICU: Intensive care unit
ID: Unique personal study number
ISO: International Organisation for Standardisation
ITT: Intention-to-treat
mRS: Modified Rankin scale
OR: Operating room
PI: Principal investigator
US: Ultrasound
US-G: Ultrasound-guided
VP: Ventriculoperitoneal
VPS: Ventriculoperitoneal shunt
